# Study on Failure of Third-Party Damage for Urban Gas Pipeline Based on Fuzzy Comprehensive Evaluation

**DOI:** 10.1371/journal.pone.0166472

**Published:** 2016-11-22

**Authors:** Jun Li, Hong Zhang, Yinshan Han, Baodong Wang

**Affiliations:** 1 College of Mechanical and Transportation Engineering, China University of Petroleum, Beijing, China; 2 PetroChina Kunlun Gas Co., LTD, Beijing, China; 3 China University of Petroleum (Beijing) Karamay Campus, Karamay, China; Beihang University, CHINA

## Abstract

Focusing on the diversity, complexity and uncertainty of the third-party damage accident, the failure probability of third-party damage to urban gas pipeline was evaluated on the theory of analytic hierarchy process and fuzzy mathematics. The fault tree of third-party damage containing 56 basic events was built by hazard identification of third-party damage. The fuzzy evaluation of basic event probabilities were conducted by the expert judgment method and using membership function of fuzzy set. The determination of the weight of each expert and the modification of the evaluation opinions were accomplished using the improved analytic hierarchy process, and the failure possibility of the third-party to urban gas pipeline was calculated. Taking gas pipelines of a certain large provincial capital city as an example, the risk assessment structure of the method was proved to conform to the actual situation, which provides the basis for the safety risk prevention.

## Introduction

As infrastructures of cities and towns, urban gas pipelines crossing streets and lanes are vulnerable to the third-party construction activities [1]. For example, an explosion occurred in a village of Beijing on April 10, 2016, causing 1 person dead, 2 people hurt and 300 square meters area burned. The reason was the rupture of gas pipeline in the process of digging [2]. According to the Study on Current Situation and Countermeasures of Urban Gas Safety Regulation System of China Gas Association, there were 1789 urban gas accidents from 2010 to 2012, and 608 of them were induced by the third-party constriction activities. The third-party damage has become a main cause of gas pipeline accidents, so it is necessary to study on the evaluation of the failure probability [3–6].

The third-party damage to urban gas pipeline is always caused by non-gas supply organization, not including natural calamities. The reasons of the third-party damage are various, leading strong randomness of occurrence of the damage, so it is hard to prevent [7]. In 2005, the United States gas group completed the gas industry research and found that nearly 35% serious incidents were caused by the third-party damage. From 1971 to 1994, among the European pipeline failure events, third party damage accounted for 32.6%, a variety of mechanical failure accounted for 25.4%, and the corrosion accounted for 30.4% [8]. FTA is an extensively used deductive analysis method to provide logical functional relationships among components and subsystems of a system by graphical description. It has been widely used in many fields, such as railway systems [9], electric power [10], and oil and gas transmission [11]. Fuzzy set theory can provide an efficient mathematical framework for estimating failure rates when available information is uncertain, incomplete, imprecise, or vague [12]. Singer [13] used a fuzzy set approach to demonstrate the relative frequencies of the basic events. Shu et al. [14] performed the failure analysis of printed circuit board assembly by integrating experts’ judgment about the possibility of failure of bottom events and by means of a triangular IFS to obtain the intuitionistic fuzzy fault tree interval. Shi et al. [15] proposed a combination of improved analysis hierarchy process (AHP) and fuzzy fault tree analysis (FFTA) to evaluated the probability of fire and explosion accidents for storage tanks (FEASOST), basing on a world-wide accident investigation of oil tanks. Lu et al. [16] performed a comprehensive risk evaluation of an underwater pipeline carrying natural gas by combining the fuzzy method with a bow-tie model. Nieto-Morote et al. [17] presented a new methodology based on the Fuzzy Set Theory and Analytic Hierarchy Process (AHP) for construction project risk analysis. Xu et al. [18] built up a third-party damage failure assessment model of urban gas pipeline network which is made up of 19 second-hierarchy and 5 first-hierarchy factors. Yang [19] proposed a mathematical model of third-party damage by fault tree analysis and fuzzy comprehensive analysis. Miao et al. [20] analyzed the failure factors of buried gas pipeline by fault tree analysis, and evaluated the failure probability and failure consequence by fuzzy comprehensive assessment method.

As mentioned above, a series of researches of the failure probability evaluation are based on the fuzzy method, but few attempted to access the failure probability of pipelines induced by the damage of third-party. In addition, the selection of risk factors in most of the previous studies on the third-party damage to gas pipelines was on the basis of statistical accident data, which is somewhat partial and not systematical. In the paper, according to the comprehensive identification on site of third party damage to the gas pipeline, a fault tree of third-party damage to urban gas pipeline was built, which contains 56 basic events. Applying fuzzy comprehensive evaluation method to the third-party damage of urban gas pipeline, not only the uncertainty of third-party damage could be reflected, but also the risk of failure can be quantified. The AHP was utilized to estimate expert ability. The evaluation of basic event probability was revised, and a group of evaluation results closer to objective probability was obtained according to the evaluation results of expert ability. The methodology proposed in this paper could provide insights for the safety evaluation for urban gas pipelines.

## Application of AHP in Determination of Expert Weight

Before introducing the evaluation methodology, the application of AHP in the determination of expert weight is proposed here. The evaluation result will be different, considering individual knowledge level, experience and perspective of experts. A model about factors which influence experts’ evaluation ability was created, and an evaluation matrix was built and the weighted correction to experts’ evaluation was carried out by AHP, according to personal situation of experts who take part in evaluation [21].

### AHP hierarchy model building

**Fig 1:**
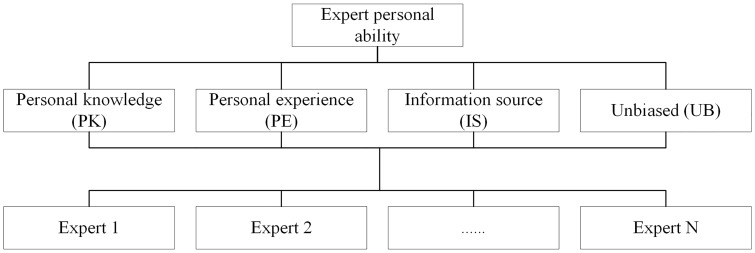
Expert ability evaluation model based AHP.

An evaluation hierarchy analysis model of expert ability evaluation was built with the following three hierarchies. The first is overall index based on experts’ individual ability. The second is sub index based on personal knowledge, personal experience, information source and unbiased. The third is each expert. The details were presented in Fig 1.

### Construction of pairwise evaluation matrix

Evaluation matrix is an expression of quantifying each hierarchy of related factors. It can adequately reflect the people’s preference among all hierarchy factors related to a certain upper hierarchy [22]. In this paper, the relative importance of each factor is quantified according to index scale.

**Step 1:** Expert ability description

Experts coming from different fields are engaged to form evaluation groups. Some are experienced, and some are solid in theoretical basis.

**Step 2:** Affecting factors ranking

The importance of each factor which influences expert evaluation ability was compared: PK = PE>IS>UB.

**Step 3:** Construction of evaluation matrix

The rule of building matrix is that when the value in matrix *C*_*ij*_ represents the superiority of *U*_*i*_ relative to that of *U*_*j*_ when comparing *U*_*i*_ with *U*_*j*_, which is met with the following relationship.

$$\{\begin{array}{c} C_{ii}=1\\ C_{ij}\cdot C_{ji}=1 \end{array}$$ (1)

Index scale was used in this paper, and details were presented in Table 1. For example, *U*_1_ is slightly important to *U*_3_, then *C*_13_ = 1.3161, and *C*_31_ = 1/1.3161 = 0.7598.

**Table 1:** Index scale.

Scale degree	Value
Equally important	1.0000
Slightly important	1.3161
Important	1.7321
Obviously important	3.0000
Strongly important	5.1966
Extremely important	9.0000

The weight effect of each index on overall index can be derived by root algorithm:

$$w_{BE,i}=\frac{\sqrt[{k}]{\overset{k}{\underset{j=1}{\prod }}c_{ij}}}{\overset{k}{\underset{m=1}{\sum }}\sqrt[{k}]{\overset{k}{\underset{j=1}{\prod }}c_{mj}}}\;i,j,m=1,2,\ldots ,k$$ (2)

Where *k* represents the index number of affecting expert evaluation ability, and *w*_*BE*,*I*_ represents the weight of index *i*, which affects expert evaluation ability.

According to index scale and the affecting factors ranking, the priority relation matrix of each index affecting the ability of expert evaluation was obtained, and the weights are calculated by Eq 2, as shown in Table 2.

**Table 2:** The priority relation matrix of each index affecting the ability of experts evaluation.

	PK	PE	IS	UB	Weight
PK	1.0000	1.0000	1.7321	3.0000	0.3453
PE	1.0000	1.0000	1.7321	3.0000	0.3453
IS	0.5773	0.5773	1.0000	1.3161	0.1861
UB	0.3333	0.3333	0.7598	1.0000	0.1233

Note: Comparing the elements of first column with each row elements of first line.

Similarly, the weight equation of different expert for a certain index is as follows.

$$w_{i,j}=\frac{\sqrt[{n}]{\overset{n}{\underset{k=1}{\prod }}{c^{\prime }}_{jk}}}{\overset{n}{\underset{k=1}{\sum }}\sqrt[{n}]{\overset{n}{\underset{k=1}{\prod }}{c^{\prime }}_{jk}}}\;i=1,2,\ldots ,k;j,k=1,2,\ldots ,n$$ (3)

Where *w*_*i*,*j*_ represents the weight of expert *j* relative to expert ability index *i*; *c’*_*jk*_ represents the superiority of expert *j* to expert *k* for the same index *i*.

Experts should be selected from different fields, such as the design, construction and installation, maintenance, management and safety of the town gas pipeline, who had no connection with the corporation to be evaluated. Comparing the personal knowledge of each expert, the priority relation matrix of personal knowledge and weight was obtained. An example of the priority relation matrix of personal knowledge and weight was shown in Table 3.

**Table 3:** The priority relation matrix of personal knowledge and weight.

	Expert 1	Expert2	Expert3	Expert4	Expert5	Expert 6	Weight
Expert 1	1.0000	1.3161	1.7321	5.1966	3.0000	1.7321	0.3014
Expert 2	0.7598	1.0000	1.3161	3.0000	1.7321	1.3161	0.2089
Expert 3	0.5773	0.7598	1.0000	1.7321	1.3161	1.0000	0.1517
Expert 4	0.1924	0.3333	0.5773	1.0000	0.7598	0.5773	0.0763
Expert 5	0.3333	0.5773	0.7598	1.3161	1.0000	0.7598	0.1100
Expert 6	0.5773	0.7598	1.0000	1.7321	1.3161	1.0000	0.1517

### Total sequencing of expert hierarchy

The expert weights can be obtained from Eq 6.

$$w_{\text{e}i}=\overset{k}{\underset{j=1}{\sum }}(w_{BE,j}\times w_{j.i})$$ (4)

where *k* is the number of evaluation index and *w*_e*i*_ is the weight of expert *i*.

## Methodology

The fuzzy evaluation on third-party damage to urban gas pipeline was carried out basing on the establishment of the fault tree. The expert evaluation method was chosen to determine the probabilities of various basic events. As it is hard to define specific values of probabilities, experts often describe the probabilities by the degree of the occurrence, such as very small, relatively small or large. The critical point is to convert these indefinite judgement to probability of occurrence. With the application of AHP and fuzzy evaluation method, the basic event probabilities were derived, and the probability of top event could be calculated accordingly.

### Establishment of a fault tree

**Fig 2:**
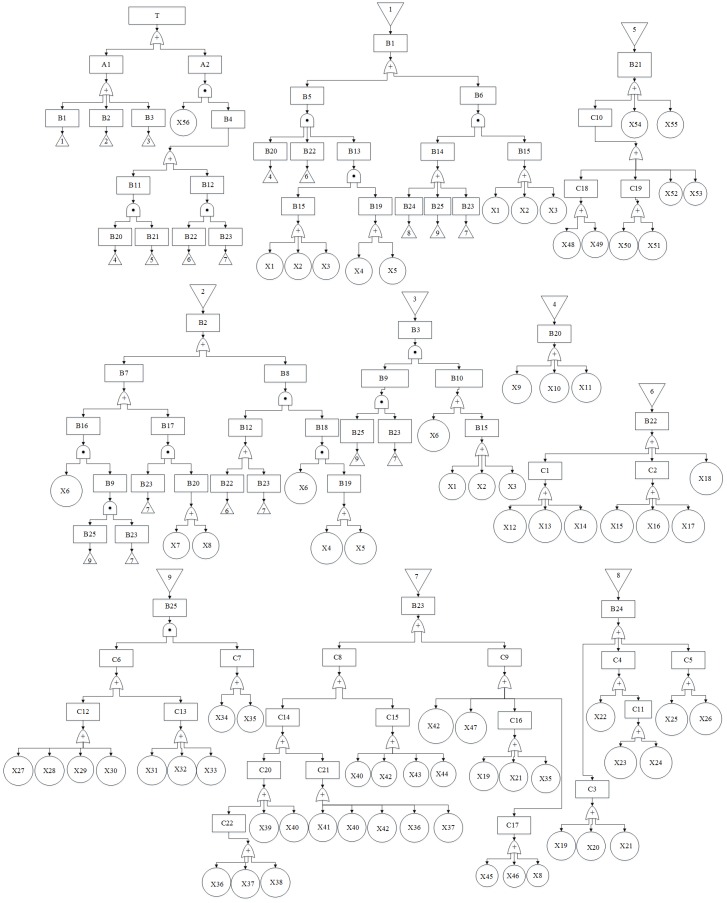
Fault tree model for third-party damage to urban gas pipeline.

The third-party damage to urban gas pipeline was selected as the top event in this paper, and all direct reasons that led to top event were chosen as intermediate events. They were connected by appropriate logic gates, such as coincidence gate and distance gate, according to the logical relationship between them [23]. Each intermediate event was analyzed from top to bottom by the same way until all the input events didn’t need to be analyzed again, obtaining all the basic events of the fault tree. The fault tree can be established by connecting the top event, intermediate events and basic events with standard notation of fault tree analysis according to the logical relationship [24]. There are 1 top event and 56 basic events of the third-party damage fault tree, as shown in Fig 2 and Table 4.

**Table 4:** The top event and basic events of the third-party damage fault tree.

Code	Event	Code	Event
T	Third-party damage to the gas pipeline	A1	Direct damage
A2	Occupied damage	B1	Pipeline damage
B2	Facilities damage	B3	Station damage
B4	Pipeline encroachment	B5	Damage by illegal construction
B6	Pipeline damage by non-illegal construction	B7	Facilities damage by non-illegal construction
B8	Facilities damage by illegal construction	B9	Mismanagement of construction site
B10	Station construction	B11	Legal defects
B12	Failure of operation and management	B13	Illegal ground-breaking
B14	Mismanagement of company	B15	Ground-breaking
B16	Damage by ground construction	B17	Non-construction damage
B18	Illegal ground construction	B19	Management flaw of constructors
B20	Law imperfection	B21	Enterprises system flaw
B22	Supervision failure	B23	Failure of signing and protection
B24	Failure of sign management	B25	Failure of on-site supervision
C1	Failure of tour inspection	C2	Defense failure
C3	Failure of pipe positioning	C4	Failure of protection scheme
C5	Failure of technical disclosure	C6	Failure of construction superintendent
C7	Failure of personnel monitoring	C8	Failure of protection
C9	Failure of signing	C10	Imperfection of institution
C11	Blocked information transmission	C12	Digging and construction
C13	Failure of backfill monitoring	C14	Failure of ground protection
C15	Failure of underground protection	C16	Wrong location of signs
C17	Breakage of signs	C18	Publicity deficiency
C19	Deficiency of rewards and penalties	C20	Fencing failure
C21	Anti-shocking failure	C22	Isolating failure
X1	Digging	X2	Piling and drilling
X3	Backfill compacting	X4	Unlicensed operation
X5	Inadequate capability	X6	Ground construction
X7	Personnel misoperation	X8	Traffic accident
X9	Lack of legal accountability	X10	Lack of enforcement
X11	Legal imperfection	X12	Imperfection of inspection system
X13	Poor responsibility of inspectors	X14	Inadequate capability of finding construction signs
X15	Disharmony with residents and property	X16	Disharmony with enterprises
X17	Disharmony with the government	X18	Incoordination of construction organization
X19	Absence of completion documents	X20	Incapability of accurate position fixing
X21	Pipeline information not updated	X22	Absence of effective protection
X23	Miscommunication of internal information	X24	Inconsistent transition of internal information
X25	No disclosure to construction unit	X26	No disclosure to onsite personnel
X27	Failure of on-site protection	X28	Misuse of construction equipment
X29	Inadequate safety distance	X30	Incompleteness of pipeline information
X31	Unsatisfactory of backfill soil	X32	Adequate buried depth
X33	Misuse of backfilling equipment	X34	Irrationality of monitoring period
X35	Poor execution of onsite personnel	X36	Inadequate interval of barriers and poles
X37	Absence of barriers	X38	Inadequate height of barriers and poles
X39	Inadequate isolation of barriers and poles	X40	Inadequate material strength
X41	Misuse of warning coloration	X42	Inadequate buffer space of anti-shocking
X43	Lack of measures of fall protection	X44	Failure of piping support
X45	Quality defect	X46	Misusing
X47	Incompleteness of warning information	X48	Lack of safety propaganda
X49	Lack of legal publicity	X50	Absence of construction monitoring incentive
X51	Inadequate punishment	X52	Lack of training
X53	Absence of reporting system	X54	Lack of leadership attention
X55	Absence of related systems to third-party damage	X56	Pipeline encroachment

### Calculation of the basic event fuzzy probability

There are many risk factors of third-party damage to urban gas pipeline, such as digging, poor responsibility of inspectors and cooperation of construction units, which are with uncertainty and randomness. In this paper, the fuzzy set theory of fuzzy mathematics was used to quantizing the factors, and the membership function of fuzzy sets were determined by trapezoidal and triangular distribution, to determine the membership function of the fuzzy set *f*(*x*), deriving the fuzzy probabilities of basic events[18].

**Step 1:** Subjective judgments about basic events

In order to reconcile both the accuracy and workload, the evaluation scope of basic events was set as: very small (VS), small(S), relatively small (RS), medium (M), relatively large (RL), large (L) and very large (VL).

**Step 2:** Quantification of expert opinion by membership function

**Fig 3:**
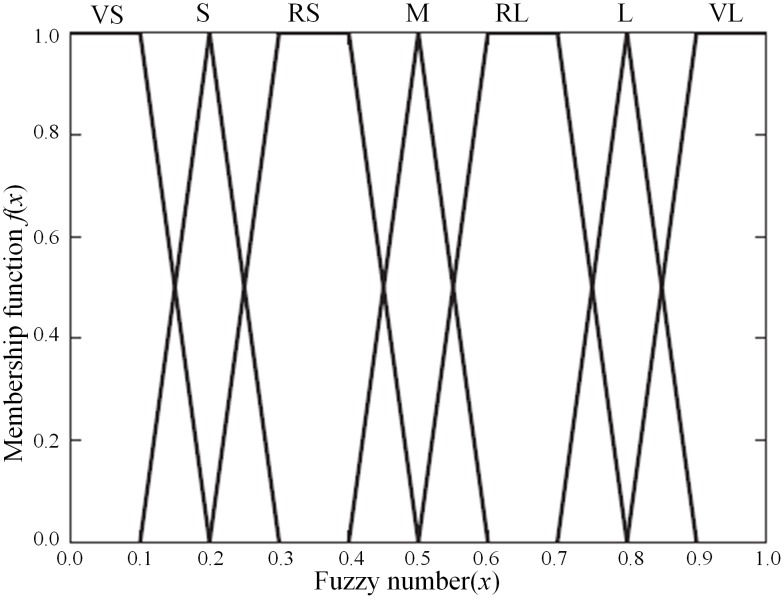
Membership function.

Expert evaluating opinions were processed by *α-*cuts of fuzzy numbers [19], obtaining the relation function of fuzzy numbers *x* corresponded to each fuzzy language (Fig 3):

$$f_{W}(x)=\{\begin{array}{cc} \frac{x-a}{0.1} & \left(a\leq x\leq a+0.1\right)\\ \frac{b-x}{0.1} & \left(b-0.1\leq x\leq b\right)\\ 1 & \left(a+0.1\leq x\leq b-0.1\right)\\ 0 & \left(\text{other cases}\right) \end{array}$$ (5)

where *a* and *b* represent the upper and lower bounds of the fuzzy number of natural language.

$$f_{\text{VS}}(x)=\{\begin{array}{c} 1.0\\ \frac{0.2-x}{0.1}\\ 0 \end{array}\;\begin{array}{c} \left(0\leq x\leq 0.1\right)\\ \left(0.1<x\leq 0.2\right)\\ \left(\text{other cases}\right) \end{array}$$ (6)

$$f_{\text{VL}}(x)=\{\begin{array}{c} \frac{0.8-x}{0.1}\\ 1.0\\ 0 \end{array}\;\begin{array}{c} \left(0.8\leq x\leq 0.9\right)\\ \left(0.9<x\leq 1.0\right)\\ \left(\text{other cases}\right) \end{array}$$ (7)

Therefore, the *α-*cuts of the fuzzy numbers are derived as follows.

$f_{\text{VS}}^{\alpha }=\left[0,0.2-0.1\alpha \right]$,$f_{\text{S}}^{\alpha }=\left[0.1+0.1\alpha ,0.3-0.1\alpha \right]$,$f_{\text{RS}}^{\alpha }=\left[0.2+0.1\alpha ,0.5-0.1\alpha \right]$,$f_{\text{M}}^{\alpha }=\left[0.4+0.1\alpha ,0.6-0.1\alpha \right]$,$f_{\text{RL}}^{\alpha }=\left[0.5+0.1\alpha ,0.8-0.1\alpha \right]$,$f_{\text{L}}^{\alpha }=\left[0.7+0.1\alpha ,0.9-0.1\alpha \right]$,$f_{\text{VL}}^{\alpha }=\left[0.8+0.1\alpha ,1\right]$.

Then the fuzzy number of one basic event can be obtained by the α-cuts integrated with the expert weight according the following steps.

$$W_{\text{w}i}=\sum_{j}^{m}w_{\text{e}j}W_{ij},\;i=1,2,\ldots ,m,\;j=1,2,\ldots ,n$$ (8)

where *W*_w*i*_ is the fuzzy number of basic event *i*, *w*_*ej*_ is the weight of expert *j*, *W*_*ij*_ is the fuzzy number evaluated by expert *j*, *m* and *n* are the total number of events and experts, respectively.

**Step 3:** Calculation of the fuzzy probability value

The fuzzy number was converted to the fuzzy probability value FPS according to the method of L-R fuzzy numbers ranking [25, 26], and the largest fuzzy set and the minimal fuzzy set was defined as follows.

$$f_{\text{max}}(x)=\{\begin{array}{c} x\\ 0 \end{array}\;\begin{array}{c} \left(0<x<1\right)\\ \left(\text{other cases}\right) \end{array}\;f_{\text{min}}(x)=\{\begin{array}{c} 1-x\\ 0 \end{array}\;\begin{array}{c} \left(0<x<1\right)\\ \left(\text{other cases}\right) \end{array}$$

The left and right fuzzy probability values of fuzzy number *W*_w*i*_ integrated with *w*_*ej*_ is:

$${\text{FPS}}_{\text{L}}\left(W_{\text{w}i}\right)=\underset{x}{\text{sup}}\left[f_{W}{}_{{}_{\text{w}i}}\left(x\right)\wedge f_{\text{max}}\left(x\right)\right]$$ (9)

$${\text{FPS}}_{\text{R}}\left(W_{\text{w}i}\right)=\underset{x}{\text{sup}}\left[f_{W}{}_{{}_{\text{w}i}}\left(x\right)\wedge f_{\text{min}}\left(x\right)\right]$$ (10)

Therefore, the fuzzy probability value of the fuzzy number *W*_w*i*_ is:

$$\text{FPS}\left(W_{\text{w}i}\right)=\left[{\text{FPS}}_{\text{R}}\left(W_{\text{w}i}\right)+1-{\text{FPS}}_{\text{L}}\left(W_{\text{w}i}\right)\right]/2$$ (11)

**Step 4:** Conversion of the fuzzy probability score (FPS) into fuzzy failure rate (FTR)

$$\text{FPR}=\left\{ \begin{array}{l} \frac{1}{10^{k}}\;\left(\text{FPS}\neq 0\right)\\ 0\;\left(\text{FPS}=0\right) \end{array}\right.$$ (12)

$$k=2.301\times {\left[\frac{1-\text{FPS}\left(W_{\text{w}i}\right)}{\text{FPS}\left(W_{\text{w}i}\right)}\right]}^{1/3}$$ (13)

The fuzzy probability value can be derived according to above steps.

### Calculation of failure probability

The final goal of the fault tree quantitative analysis is to obtain the probability of the top event. The failure probability of gas pipeline system induced by third-party damage, which was quantitatively evaluated according to the fault tree. The quantitative analysis principle is as follows. Assuming that *x*_*i*_ is the occurrence probability of basic event *i*, and *Y* is the occurrence probability of top event.

The probability of fault tree can be described by minimal cut sets which are connected by distance gates, and the probability function is:

$$Y^{or}=1-\prod_{i=1}^{n}\left(1-x_{i}\right)$$ (14)

According to exponential scale tables, the matrix of expert weight was obtained by the method of AHP and the matrix of expert ability weight. Then the weight calculation of evaluation results on some certain pipelines given by experts was accomplished, and the failure probability of pipelines was derived.

## Fuzzy Evaluation of an Urban Gas Pipeline

A medium pressure urban gas pipeline of Harbin Zhongqing Gas Company, Heilongjiang Province was selected as a calculation example. The PE pipeline is located in the center of the city, with the length of 7.2km, the actual average depth of 1.7m and the pressure of 0.28MPa.

### Determination of the weight of expert ability

The 6 experts of the evaluation group were selected from different fields, such as the design, construction and installation, maintenance, management and safety of the town gas pipeline, who had no connection with the corporation to be evaluated. The priority relation matrix of personal knowledge and weight was set as the same as given in Section of Construction of pairwise evaluation matrix, and the rest matrixes, i.e., the priority relation matrix of expert personal experience, information source and unbiased were shown in Tables 5–7.

**Table 5:** The priority relation matrix of personal experience and weight.

	Expert 1	Expert2	Expert3	Expert4	Expert5	Expert 6	Weight
Expert 1	1.0000	1.3161	3.0000	0.7598	1.7321	1.3161	0.2124
Expert 2	0.7598	1.0000	1.0000	0.5773	1.3161	1.0000	0.1407
Expert 3	0.3333	1.0000	1.0000	0.1924	1.0000	0.7598	0.0932
Expert 4	1.3161	1.7321	5.1966	1.0000	3.0000	1.3161	0.2927
Expert 5	0.5773	0.7598	1.0000	0.3333	1.0000	0.7598	0.1069
Expert 6	0.7598	1.0000	1.3161	0.7598	1.3161	1.0000	0.1542

**Table 6:** The priority relation matrix of information source and weight.

	Expert 1	Expert2	Expert3	Expert4	Expert5	Expert 6	Weight
Expert 1	1.0000	0.7598	1.3161	1.7321	3.0000	1.3161	0.2122
Expert 2	1.3161	1.0000	1.7321	3.0000	5.1966	1.3161	0.2924
Expert 3	0.7598	0.5773	1.0000	1.0000	1.3161	1.0000	0.1406
Expert 4	0.5773	0.3333	1.0000	1.0000	1.0000	0.7598	0.1118
Expert 5	0.3333	0.1924	0.7598	1.0000	1.0000	0.7598	0.0889
Expert 6	0.7598	0.7598	1.0000	1.3161	1.3161	1.0000	0.1540

**Table 7:** The priority relation matrix of personal unbiasedness and weight.

	Expert 1	Expert2	Expert3	Expert4	Expert5	Expert 6	Weight
Expert 1	1.0000	0.7598	1.7321	1.3161	0.5773	1.7321	0.1652
Expert 2	1.3161	1.0000	1.7321	1.7321	0.7598	3.0000	0.2174
Expert 3	0.5773	0.5773	1.0000	0.7598	0.3333	1.3161	0.1045
Expert 4	0.7598	0.5773	1.3161	1.0000	0.5773	1.7321	0.1375
Expert 5	1.7321	1.3161	3.0000	1.7321	1.0000	5.1966	0.2995
Expert 6	0.5773	0.3333	0.7598	0.5773	0.1924	1.0000	0.0759

By the calculation procedure introduced in Section of Total sequencing of expert hierarchy, the expert weights were calculated below: *w*_e_ = (0.2373, 0.2019, 0.1236, 0.1652, 0.1284, 0.1436).

### Evaluation of risk factors

56 basic events in Section of Methodology were selected as risk factors to compile the assessment questionnaire. 6 experts filled the evaluation form based on the field investigation and personal experience and knowledge, as shown in Table 8.

**Table 8:** Expert assessment statistics.

Number	Risk factors	Expert Scoring					
		Expert 1	Expert 2	Expert 3	Expert 4	Expert 5	Expert 6
1	Digging	VS	VL	VL	VL	VL	VL
2	Piling	RS	M	RL	L	L	M
3	Drilling	M	L	L	L	L	L
4	Unlicensed operation	VL	L	VL	VL	RL	VL
5	Inadequate capability	VL	RL	VL	VL	VL	VL
6	Ground construction	VS	RS	VS	S	RS	M
7	Personnel misoperation	M	VL	RL	M	M	M
8	Traffic accident	VS	L	VS	S	VS	S
9	Lack of legal accountability	RS	L	VS	VL	VL	VL
10	Lack of enforcement	RL	VL	VL	VL	VL	VL
11	Legal imperfection	M	L	VL	VL	VL	L
12	Imperfection of inspection system	L	VL	L	VL	VL	RL
13	Poor responsibility of inspectors	VL	VL	VL	VL	VL	L
14	Inadequate capability of finding construction signs	L	L	L	VL	VL	L
15	Disharmony with residents and property	M	RL	M	VL	M	RS
16	Disharmony with enterprises	M	RL	M	VL	M	RS
17	Disharmony with the government	L	RL	M	VL	M	S
18	Incoordination of construction organization	VL	VL	VL	VL	L	VL
19	Absence of completion documents	L	VL	L	RL	L	VL
20	Incapability of accurate position fixing	VL	VL	VL	RL	RL	VL
21	Pipeline information not updated	VL	VL	L	VL	M	RL
22	Absence of effective protection	L	L	L	L	M	M
23	Miscommunication of internal information	L	L	VL	L	L	M
24	Inconsistent transition of internal information	L	L	VL	L	L	M
25	No disclosure to construction unit	L	L	VL	L	L	RS
26	No disclosure to onsite personnel	VL	L	VL	M	L	M
27	Failure of on-site protection	VL	VL	L	M	M	VL
28	Misuse of construction equipment	VL	RL	RL	VL	L	VL
29	Inadequate safety distance	L	RL	L	L	L	L
30	Failure of support	RL	L	VL	VL	VL	VL
31	Unsatisfactory of backfill soil	M	RL	RL	RL	RL	RL
32	Adequate buried depth	L	VL	RL	VL	VL	VL
33	Misuse of backfilling equipment	L	M	RL	RS	RL	M
34	Irrationality of monitoring period	RL	L	L	VL	VL	VL
35	Poor execution of onsite personnel	VL	VL	VL	VL	M	VL
36	Inadequate interval of barriers and poles	VS	M	RL	M	M	M
37	Absence of barriers	VS	RL	RL	M	M	M
38	Inadequate height of barriers and poles	VS	M	M	M	M	M
39	Inadequate isolation of barriers and poles	VS	M	L	M	M	RS
40	Inadequate material strength	RS	RL	RL	M	M	RS
41	Misuse of warning coloration	VS	RL	RS	M	M	RS
42	Inadequate buffer space of anti-shocking	RS	M	RL	M	M	M
43	Lack of measures of fall protection	M	L	RL	M	M	S
44	Failure of piping support	M	L	RL	RS	RS	S
45	Quality defect	VS	L	L	VL	L	S
46	Misusing	M	L	RL	L	L	S
47	Incompleteness of warning information	S	RL	RL	M	L	RS
48	Lack of safety propaganda	M	L	L	M	M	L
49	Lack of legal publicity	RL	L	VL	M	M	L
50	Absence of construction monitoring incentive	RL	L	L	L	RS	M
51	Inadequate punishment	L	L	L	RL	RS	RL
52	Lack of training	M	L	L	L	L	RL
53	Absence of reporting system	M	RL	L	RL	M	M
54	Lack of leadership attention	M	VL	VL	VL	VL	RL
55	Absence of related systems to third-party damage	M	VL	VL	L	L	L
56	Pipeline encroachment	M	L	M	VL	RL	L

### Failure probability of the pipeline

The expert fuzzy evaluation was quantified by using the theory of fuzzy mathematics introduced in Section of Methodology, deriving the fuzzy probabilities of basic events of relevant pipes. Take the basic event of digging for example. According to the expert scoring on it, i.e., VS, VL, VL, VL, VL and VL, the fuzzy number of the basic event integrated with expert weight *W*_w*i*_ can be described as follows.

$$\begin{array}{l} f\left(x\right)=\text{max}\left[w_{\text{e}1}f_{\text{VS}}\left(x\right)\wedge w_{\text{e2}}f_{\text{VL}}\left(x\right)\wedge w_{\text{e3}}f_{\text{VL}}\left(x\right)\wedge w_{\text{e4}}f_{\text{VL}}\left(x\right)\wedge w_{\text{e5}}f_{\text{VL}}\left(x\right)\wedge w_{\text{e6}}f_{\text{VL}}\left(x\right)\right]\\ \;=[0.2373\times 0+0.2019\times \left(0.8+0.1\alpha \right)+0.1236\times \left(0.8+0.1\alpha \right)+\ldots +0.1436\times \\ \;\left(0.8+0.1\alpha \right),0.2373\times \left(0.2-0.1\alpha \right)+0.2019\times 1+0.1236\times 1+\ldots +0.1436\times 1]\\ \;=\left[\left(0.07627\alpha +0.61016\right),\left(0.81016-0.02373\alpha \right)\right] \end{array}$$

**Fig 4:**
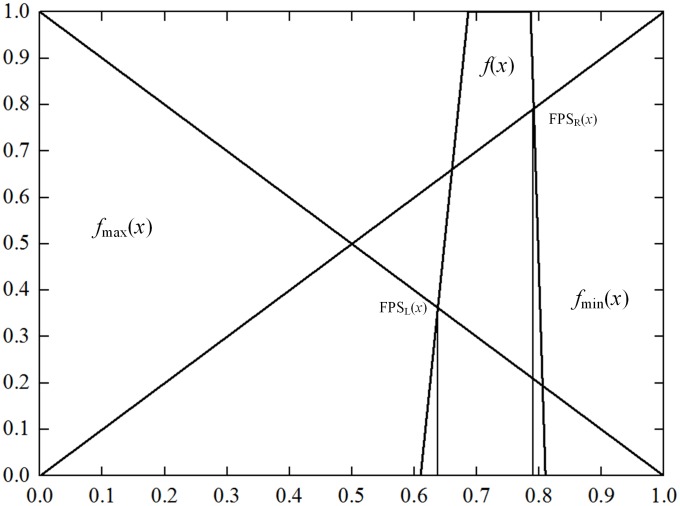
Membership function of x1.

Therefore, the expert weight integrated function of the fuzzy number *x* (Fig 4) can be described as:

$$f(x)\;=\;\{\begin{array}{ll} \frac{x-0.61016}{0.07627} & 0.61016<x\leq 0.68643\\ 1 & 0.68643<x\leq 0.78643\\ \frac{0.81016-x}{0.02373} & 0.78643<x\leq 0.81016\\ 0 & \text{other cases} \end{array}$$ (15)

The values of FPS and FPR are as follows: FPSL(x) = 0.36221, FPSR(x) = 0.79138, FPS(x) = 0.714585 and FPR = 0.020207. So the failure rate of digging is 0.020207.

As the calculation is tedious, the MATLAB software can be utilized. After the calculation of failure rates of all basic events, the failure probability of the gas pipeline was derived to be 1.55%. From site record, Harbin Zhongqing Gas Company has 1958 km gas pipeline, occurred 23 accidents of third-party damage in 2015, as shown in Table 9. The number of accidents to third-party damage is 0.0117 / (km∙a^-1^). By referring to related accident records of the gas pipeline, it was generally consistent with result of high failure. Therefore, some measures should be taken to reduce the risk of the third-party damage to pipelines.

**Table 9:** Third-party damage to gas pipeline accidents in 2015.

Date	accident type
4.4	accident of Middle-pressure PE gas pipeline diameter 200 mm occurred in Pingfang Road
5.1	accident of PE gas pipeline diameter 50 mm occurred in Mulan Road
5.25	accident of PE gas pipeline diameter 75 mm occurred in Lianbu Road
5.31	accident of PE gas pipeline diameter 63 mm occurred in Caoshi Road
7.14	accident of PE gas pipeline diameter 60 mm occurred in Wenzheng Road
7.18	accident of PE gas pipeline diameter 63 mm occurred in Lanqin Road
7.22	accident of PE gas pipeline diameter 63 mm occurred in Huaihe Road
7.22	accident of Middle-pressure PE gas pipeline diameter 110 mm occurred in Aijian Road
7.25	accident of PE gas pipeline diameter 63 mm occurred in Anning Road
7.29	accident of PE gas pipeline diameter 75 mm occurred in Lanyin Road
8.1	accident of Middle-pressure PE gas pipeline diameter 160 mm occurred in Haxi Road
8.10	accident of Middle-pressure PE gas pipeline diameter 110 mm occurred in Longdan Road
8.17	accident of PE gas pipeline diameter 63 mm occurred in Dongan Road
9.3	accident of Middle-pressure PE gas pipeline diameter 125 mm occurred in Xinjiang Road
9.8	accident of PE gas pipeline diameter 63 mm occurred in Zhongshan Road
9.20	accident of PE gas pipeline diameter 63 mm occurred in Nanma Road
9.23	accident of Middle-pressure PE gas pipeline diameter 63 mm occurred in Shuini Road
10.7	accident of PE gas pipeline diameter 63 mm occurred in Xinle Road
10.10	accident of PE gas pipeline diameter 63 mm occurred in Xuanhua Road
11.4	accident of Middle-pressure PE gas pipeline diameter 50 mm occurred in Mucai Road
11.7	accident of PE gas pipeline diameter 63 mm occurred in Xianfeng Road
11.17	accident of PE gas pipeline diameter 60 mm occurred in Xuanhua Road
12.8	accident of PE gas pipeline diameter 63 mm occurred in Xicai Road

## Conclusions

A failure evaluation of third-party damage for urban gas pipelines was conducted in this paper, combing AHP and fuzzy comprehensive evaluation method. Conclusions can be drawn as follows:

1. The fault tree which top event was the third-party damage to urban gas pipeline was systematically established in this paper, which contains 56 basic events.
2. Based on the fuzzy set theory, 56 risk factors of the third-party damage to urban gas pipelines were quantified to compute the fuzzy probabilities.
3. A hierarchy model of influence factors of abilities of expert evaluation was built by the improved analytic hierarchy process. The matrix of evaluation was built according to the personal information of experts, and the expert evaluation was weighting modified by AHP, whose results were more close to the objective probability.
4. Based on the method of comprehensive fuzzy evaluation, the failure probability was derived by practical application, which was in agreement with the statistical data of the gas company, verifying the feasibility and effectiveness of evaluation methods and models.
